# Prevalence of antibiotic-resistant *E. coli* in retail chicken: comparing conventional, organic, kosher, and raised without antibiotics

**DOI:** 10.12688/f1000research.2-155.v2

**Published:** 2013-09-02

**Authors:** Jack M Millman, Kara Waits, Heidi Grande, Ann R Marks, Jane C Marks, Lance B Price, Bruce A Hungate

**Affiliations:** 1 Horace Mann Bronx Campus, Bronx NY, 10471, USA; 2 Translational Genomics Research Institute, Flagstaff AZ, 86001, USA; 3 Department of Biological Sciences and Merriam-Powell Center for Environmental Research, Northern Arizona University, Flagstaff AZ, 86011, USA; 4 Department of Environmental and Occupational Health, George Washington University, Washington DC, 20037, USA

## Abstract

Retail poultry products are known sources of antibiotic-resistant *Escherichia coli*, a major human health concern. Consumers have a range of choices for poultry, including conventional, organic, kosher, and raised without antibiotics (RWA) – designations that are perceived to indicate differences in quality and safety. However, whether these categories vary in the frequency of contamination with antibiotic-resistant *E. coli* is unknown. We examined the occurrence of antibiotic-resistant *E. coli* on raw chicken marketed as conventional, organic, kosher and RWA. From April – June 2012, we purchased 213 samples of raw chicken from 15 locations in the New York City metropolitan area. We screened *E. coli* isolates from each sample for resistance to 12 common antibiotics. Although the organic and RWA labels restrict the use of antibiotics, the frequency of antibiotic-resistant *E. coli* tended to be only slightly lower for RWA, and organic chicken was statistically indistinguishable from conventional products that have no restrictions. Kosher chicken had the highest frequency of antibiotic-resistant *E. coli*, nearly twice that of conventional products, a result that belies the historical roots of kosher as a means to ensure food safety. These results indicate that production methods influence the frequency of antibiotic-resistant *E. coli *on poultry products available to consumers. Future research to identify the specific practices that cause the high frequency of antibiotic-resistant *E. coli* in kosher chicken could promote efforts to reduce consumer exposure to this potential pathogen.

## Introduction

The use of antibiotics in livestock production may pose health risks to humans, as such usage has been correlated with the occurrence of antibiotic-resistant bacteria isolated from human infections ^1, 2^. Methods of livestock production differ in antibiotic use, and this can influence the frequency of antibiotic-resistant bacteria on retail meats. For example, antibiotic-resistant *Escherichia coli* has been shown to be less common on poultry raised without antibiotics (RWA) as compared to poultry raised conventionally ^3^. Likewise, organic poultry can have lower frequencies of antibiotic-resistant bacteria than poultry raised conventionally ^4– 10^, although this is not always the case ^11– 13^. Organic, RWA, and kosher food products supply a growing market niche ^14^. Consumers perceive that they offer health benefits ^14– 21^ and are willing to pay a premium for them ^22– 24^. The actual health benefits of organic food are not always clear ^25^, and the health benefits of kosher foods are largely anecdotal. Little is known about the frequency of antibiotic-resistant microorganisms on kosher products.

The organic and RWA labels require specific production methods as stipulated in US federal regulations, whereas the kosher label adheres to religious requirements that are regulated privately. The RWA label requires that “livestock have never received antibiotics from birth to harvest” ^26^. The United States Department of Agriculture (USDA) organic standard is only slightly less strict, stipulating that “The producer of an organic livestock operation must not sell, label, or represent as organic any animal or edible product derived from any animal treated with antibiotics”, but also that “Poultry or edible poultry products must be from poultry that has been under continuous organic management beginning no later than the second day of life” ^26, 27^. Therefore, injecting antibiotics into eggs or administering them during the first 24 hours of the chick’s life will not violate the letter of the USDA organic standard ^28, 29^. Kosher production differs from organic and RWA in that it is inherently predicated on religious requirements. For kosher meat, the major requirements are that it must be from animals that have split hooves and chew their cud, it must not be mixed with dairy products, and all equipment used must be used exclusively for kosher food ^19^. Animals must be slaughtered “humanely”, and meat is typically salted to remove blood rapidly, a practice that has been shown to reduce the microbial load ^30^. Unlike for organic and RWA, kosher poultry is not regulated by Federal laws but rather by private certification organizations, and thus the specific practices vary ^19^.

Here, we compared four major types of poultry-conventional, kosher, organic, and RWA-in order to assess the frequency of contamination with antibiotic-resistant *E. coli*. We focused on poultry products from a major metropolitan center (the greater New York City area) and products available to typical consumers by studying multiple brands of chicken from multiple stores. Our goal was to compare the frequency of antibiotic-resistant *E. coli* in these four categories of chicken.

## Methods

### Sample collection

During April–June 2012, raw chicken was purchased from supermarkets, butcher shops, specialty stores, and food distributors in the greater New York City area. A variety of widely available brands were procured in four categories: conventional, kosher, organic and RWA. Within each category of chicken purchased, we collected at least four samples of each brand. Some samples included more than one category (e.g., kosher and organic). Five collections occurred resulting in 213 total samples. Samples were drumsticks or samples from which drumsticks were removed for analysis (all with skin). After purchase, each chicken sample was placed in a labeled, ziplock bag, and placed in a cooler with ice packs. Three coolers with ice packs were shipped overnight to T-Gen North within two days of collection.

### Laboratory analyses

Chicken samples arrived at the laboratory in their original packaging and were refrigerated at 4°C until processed. One putative *E. coli* strain was isolated and screened from each sample using standard methods for assaying for antimicrobial resistance described by the Clinical and Laboratory Standards Institute (CLSI) ^31^. The use of one strain per sample enabled efficient testing among a population of chicken samples for differences in the frequency of antibiotic resistance.

One whole drumstick was selected from each package or removed from each whole chicken sample using a sterilized knife. Each sample was transferred aseptically to a Stomacher Bag (VWR, Radon, PA, USA, catalog number 11216–902) containing 250 ml MacConkey broth (Alpha Biosciences, Baltimore, MD) and agitated at speed 7 for 3 min on a rocking platform shaker (VWR, Radon, PA, USA, model no. 40000–302) and incubated overnight at 44°C. A 10 μl loop was used to inoculate a VRBA+MUG (Teknova, Hollister, CA) plate with the enriched broth. The plate was incubated at 37°C for 2 h and then at 44°C for 22 h, along with QA/QC strains ATCC *E. coli* 35218, *Klebsiella pneumoniae*, *Hafnia alvei*, *Citrobacter freundii* and *Serratia plymuthica.* QA/QC strains not listed as ATCC were isolated and identified using the BD Phoenix at Flagstaff Medical Center. From each VRBA+MUG plate, four putative *E. coli* colonies were streaked to CHROMagar (Hardy Diagnostics, Santa Maria, CA) and incubated 20 to 24 h at 37°C. One putative *E. coli* colony, appearing pink to rose, was streaked to a second CHROMagar plate and incubated 20 to 24 h at 37°C. For each sample, a putative *E. coli* isolate was inoculated into an assigned well of a 96-well plate containing 75 µl of Tris EDTA (TE) buffer. DNA was released from cell suspension with a thermal cycler (Bio-Rad, Hercules, CA) using the following parameters: heated lid, 95°C; block temperature, 90°C for 15 min. To confirm the identity of putative *E. coli* isolates, a *uidA* qPCR assay and a universal bacterial qPCR (BactQuant ^32^) were used. For each reaction, 2 μl of DNA was added into 8 μl of master mix, with the final reaction containing 1.8 μM of each forward and reverse *uidA* primer, 0.25 μM *uidA-* VIC probe, 0.90 μM of each forward and reverse Pan16S primer, 0.25 μM Pan16S-FAM probe, 1X QuantaPerfeCTa ^®^ Multiplex qPCR SuperMix w⁄ROX (Quanta Biosciences, Gaithersburg, MD) and molecular-grade water. All samples were run in triplicate and each experiment included a standard curve and no-template controls. The 7900HT Real-Time PCR System (Applied Biosystems, Carlsbad, CA) was used to run the reactions with following conditions: 3 min at 50°C for UNG treatment, 10 min at 95°C for *Taq* activation, 15 s at 95°C for denaturation and 1 min at 60°C for annealing and extension × 40 cycles. Six isolates were excluded from further analysis because they were not confirmed as *E. coli* using the qPCR assay.

Guidelines from the Clinical and Laboratory Standards Institute (CLSI) for disk diffusion methods ^31^ were used to test each strain for resistance to antibiotics. Some strains did not grow under assay conditions (n=23) and were excluded from further analysis. Twelve antibiotics were tested, representing seven classes of drugs: tetracycline (class, tetracyclines); ampicillin and ampicillin sulbactam (class, penicillins); cefazolin, cefoxitin, and ceftriaxone (class, cephalosporins); gentamicin and amikacin (class, aminoglycosides); nalidixic acid and ciprofloxacin (class, quinolones); trimethoprim sulfamethoxazole (class, folate pathway inhibitors); and imipenem (class, carbapenems) (VWR, Radon, PA). Breakpoint guidelines from the CLSI M100 Tables 2A through 2J for *E. coli* ^31^ were used to classify strains into “resistant”, “intermediate” or “susceptible”; designations of “intermediate” were lumped with “resistant” for purposes of statistics and inference, a conservative approach with respect to consumer safety.

### Statistical analyses

Analysis of variance (ANOVA) was used to test whether antibiotic resistance varied among the brands of chicken sampled, using SYSTAT 13.1. Effects of brand within each category were tested (i.e., using all the data within conventional, organic, kosher, RWA). For each drug, Microsoft Excel for Mac Version 14.1.0 was used to conduct chi-square tests to determine whether the frequency of resistance varied among categories of chicken: conventional, organic, kosher and RWA.

The total number of drugs and drug classes to which each strain was resistant were enumerated. One-way ANOVA was used to compare the average number of drugs to which strains were resistant among categories, using samples with only one category designation (n=120). This test captures the effect of a consumer’s choice whether to purchase chicken in one category over another on the likelihood of exposure to antibiotic-resistant *E. coli*.

Multi-factor ANOVA was used to test whether trends held across the broader dataset (n=184), including samples with multiple category designations. The collection of samples included adequate replication (>14) for every possible two-way combination of labels (organic & kosher, RWA & organic, and RWA & kosher). Replication for the three-way combination (organic, kosher & RWA) was low (n=5), and all samples were from one brand. To avoid bias, these samples were excluded from the ANOVA. Each of the three labeling categories was included as a factor in three-way ANOVAs (organic, RWA, and kosher, each with two levels), with the number of drugs and drug classes exhibiting resistance as response variables. This tests for the effect of each category and for interactive effects of combining categories.

## Results

Across the entire dataset, resistance to cefazolin was most common (41.3%), followed by ampicillin (31.5%), tetracycline (30.4%), and ampicillin sulbactam (19.6%). Some resistance was detected for cefoxitin, (12.5%) and gentamicin (10.9% of strains), but no strain was resistant to amikacin, the other aminoglycoside tested. For the quinolones, some (3.3%) of strains were resistant to nalidixic acid, but none was resistant to ciprofloxacin. Resistance was low (3.3%) for trimethoprim sulfamethoxazole, the one folate pathway inhibitor tested, and was absent for imipenem, the one carbapenem tested. Over half of all strains collected exhibited resistance to one or more antibiotics: 55%, 58%, 60%, and 76% from conventional, RWA, organic, and kosher chicken samples, respectively.

**Figure 1.**
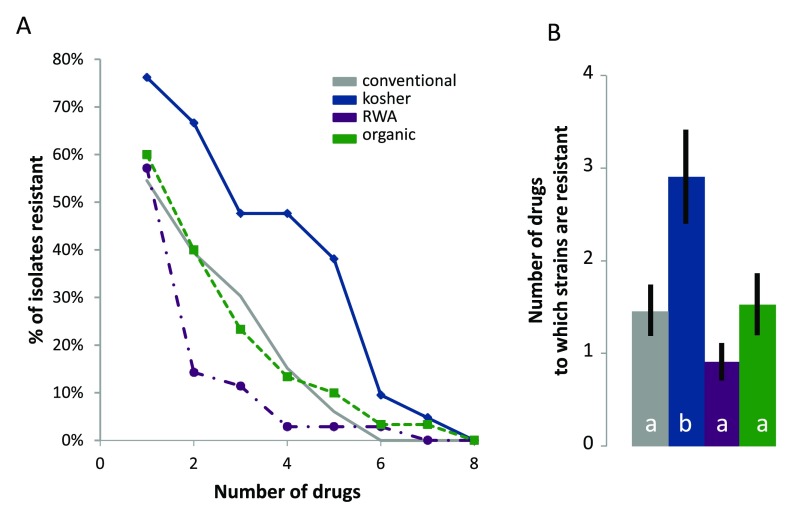
**A**. The percentage of resistant strains of *E. coli* as a function of the number of drugs tested for each of the four categories of chicken sampled. Values shown on the x-axis are cumulative. For example, the percentage of strains resistant to five or more drugs includes strains resistant to five to seven drugs. **B**. The average number of drugs to which strains of *E. coli* exhibited resistance in each of the four categories of chicken sampled. Values shown are means ± standard errors of the mean. Category was a significant factor in a one-way ANOVA (P=0.003). Bars with different letters are significantly different at P<0.05 (Tukey’s HSD). RWA-raised without antibiotics.

Within categories of chicken purchased, brands did not vary in the extent of antibiotic resistance ( Table 1). By contrast, categories of chicken differed in the number of drugs to which strains of *E. coli* were resistant ( Figure 1). Strains of *E. coli* isolated from kosher chicken were resistant to more drugs than were strains from the other categories (Tukey’s HSD comparisons: kosher vs. conventional, P=0.023; kosher vs. organic, P=0.041; kosher vs. RWA, P=0.002).

**Table 1.** Results from four one-way ANOVA testing for the effect of brand on *E. coli* drug resistance. The response variable was the number of drugs to which strains of *E. coli* exhibited resistance. N indicates numbers of brands within each category. The P-values are for the effect of brand, tested for each category.

Category	N	P-value
Conventional	9	0.129
Organic	13	0.367
Kosher	10	0.789
RWA	14	0.607

**Figure 2.**
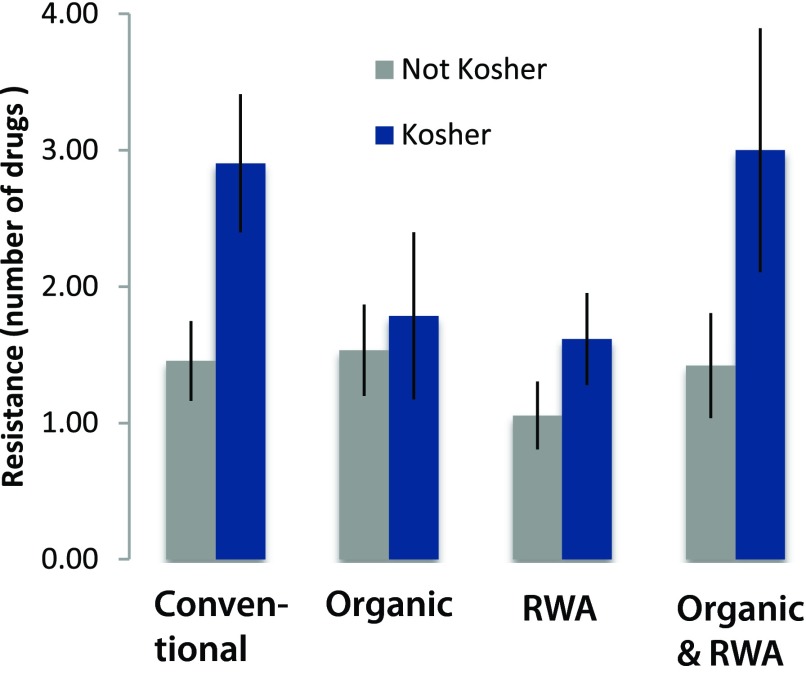
Antibiotic resistance across all categories tested, showing the number of drugs to which strains of *E. coli* were resistant among categories. Values shown are means ± standard errors of the mean. Kosher was a significant factor in the analysis of variance (P=0.00374), whereas ‘raised without antibiotics’ (RWA) (P=0.122), organic (P=0.874), and all interactions (P<0.050) were not significant.

These patterns held when analyzing the broader dataset, including the samples with multiple designations. Strains of *E. coli* isolated from kosher chicken samples were resistant to more drugs compared to the other categories ( Figure 2). Strains of *E. coli* isolated from samples in the RWA category tended to be resistant to fewer drugs but the difference was not significant versus conventional and organic which did not differ from each other.

Laboratory assay assessing antibiotic resistance in isolates of Escherichia coli from retail chicken collected in the New York metropolitan area Results from the disk diffusion test. Metric shown is the distance (in mm) of edge of growth lawn from edge of disk. Lower numbers indicate greater resistance. 'Total' refers to the total number of drugs to which strain is resistant or intermediate.

## Discussion

Poultry growers use antibiotics both for therapeutic purposes and for growth promotion ^33, 34^. Based on a national survey conducted by the USDA of poultry and hog producers in the United States, use of antibiotics at sub-therapeutic levels for growth promotion is common ^35, 36^. One estimate places growth promotion in livestock production as the single largest sector in which antibiotics are used in the US, accounting for 70% of the total of 50 million pounds for the year 2008 ^37^. The use of antibiotics in poultry production can select for antibiotic-resistant microorganisms including *Salmonella*, *Campylobacter*, *Enterococcus*, and extra-intestinal pathogenic *E. coli* ^38^. Studies of *E. coli* from bloodstream infections in Europe suggest that poultry are an important source of antibiotic-resistant infections ^39^. Use of antibiotics is restricted in production of chicken carrying the USDA organic and USDA RWA labels. Like conventional chicken, chicken with a certified kosher label does not indicate any special restrictions in the use of antibiotics.

Our finding that brands within categories did not differ significantly in the extent of antibiotic resistant *E. coli* ( Table 1) could arise from the fact that individual brands of chicken obtain product from multiple farms whose production practices may differ, obscuring clear patterns associated with individual brands. Our ability to detect an effect of brand might also be constrained by low statistical power. Our finding that the frequency of antibiotic resistant strains of *E. coli* on organic poultry did not differ significantly from conventional ( Figure 1 and Figure 2) reflects some past studies in this area that have found no difference in antibiotic resistance between organic and conventional practices ^11– 13^. Others found that pathogens on organic or RWA poultry products had lower resistance to antibiotics compared to conventional products ^4, 10, 40– 43^, which was the trend we observed for RWA. The distinction between USDA organic from USDA RWA may be important, given that organic chicks can receive antibiotics via *in ovo* injections and during the first day of life. Previous studies have provided unequivocal evidence that even *in ovo* injection of antibiotics can affect the susceptibility of the bacteria that contaminate poultry products ^2^. With a larger sample, the tendency for *E. coli* isolated from RWA samples to have lower frequency of antibiotic resistance than other categories (P=0.122; Figure 1 and Figure 2) may emerge as significant.

Cross-contamination is another possible source of antibiotic resistance ^44^. Shared facilities for product and slaughter could promote cross-contamination and antibiotic strains could be spread among organism and environments ^45, 46^. Poultry could then be inadvertently exposed to antibiotic-resistant *E. coli*. For example, companies with both conventional and organic products may slaughter in the same facilities, promoting cross-contamination. Production facilities that convert from one practice to another could also experience residual contamination, though there is evidence that converting from conventional to organic can reduce frequency of resistance ^8^. The identification of possible cross-contamination is outside the scope of this study, but these possibilities would need to be considered when investigating the sources of antibiotic resistance.

The increased resistance of *E. coli* in kosher chicken compared to conventional was surprising, because, while kosher does not stipulate anything about antibiotic use, kosher is perceived as clean and safe to consume ^19^. The higher resistance found in isolates from kosher chicken ( Figure 1 and Figure 2), and the distinct antibiotic-resistance profile ( Table 2) suggests that use of antibiotics in the kosher production chain is common and that it may be more intensive than use of antibiotics among conventional, organic, or RWA practices. It is not immediately obvious where in the kosher chicken production process antibiotic use might be more prevalent, or where exposure to antibiotic-resistant organisms is more likely. Consumers perceive organic, kosher and RWA products to be healthier ^14– 21^, though the real health benefits from organic products are unclear ^10^, and, to our knowledge, the actual health benefits of kosher have not been assessed. Our findings are consistent with the suggestion that some ‘niche market’ products, while perceived to be safer, may have higher incidence of foodborne pathogens compared to conventional products ^47^.

**Table 2.** Antibiotic-resistance profiles of conventional, organic, kosher and ‘raised without antibiotics’ (RWA) chicken products. Bold text denotes significant differences among categories according to one-way ANOVA.

Antibiotic	Conventional	Organic	Kosher	RWA	P-value
Ampicillin	24%	33%	62%	14%	**0.002**
Ampicillin sulbactam	18%	13%	52%	8%	**0.001**
Cefazolin	30%	43%	62%	31%	0.072
Cefoxitin	3%	10%	33%	6%	**0.003**
Ceftriaxone	3%	7%	33%	6%	**0.001**
Nalidixic acid	3%	3%	5%	3%	0.981
Gentamicin	24%	13%	5%	11%	0.206
Tetracycline	30%	30%	33%	25%	0.917
Trimethoprim sulfamethoxazole	9%	0%	5%	3%	0.321

Our study was limited in geographic and temporal scale, as we focused on the New York metropolitan area over a three-month time period. Yet, the region is large and populous, we focused on the most widely available brands in all categories, and this area particularly offered multiple kosher brands. Our final sample size was limited (n=184) but not atypical for the field ^48– 51^. Finally, we only assayed for generic *E. coli* and did not assess virulence or virulence group assignments for each sample. However, *E. coli* is a useful focal organism because it is widespread and an important potential pathogen.

More studies are needed to test whether antibiotic resistance among kosher products is consistently higher than conventional and other categories. Nevertheless, our study offers insight into another area of the food production system increasing the exposure of people to microorganisms that are resistant to antibiotics. In addition to regulation, more consistent surveillance or auditing would add of consumer protection, enabling improved purchase decisions based on price and health benefits guided by meaningful labels.
