# Supplementary material for: Prevalence of antibiotic-resistant E. coli in retail chicken: comparing conventional, organic, kosher, and raised without antibiotics
Source: F1000Res. 2013 Sep 2;2:155. Originally published 2013 Jul 11. [Version 2] doi: 10.12688/f1000research.2-155.v2 (PMC3901448; doi:10.12688/f1000research.2-155.v2)
Supplement: Peer review history

# Peer review: Referee response for version 2

**Published:** 2013-09-17

**Authors:** Irene Hanning

**Affiliations:** 1 Food Science and Technology, University of Tennessee, Logan, TN, USA

All previous comments have been addressed by the authors.

I have read this submission. I believe that I have an appropriate level of expertise to confirm that it is of an acceptable scientific standard.

# Peer review: Referee response for version 2

**Published:** 2013-09-09

**Authors:** Katharina Stärk

**Affiliations:** 1 Veterinary Epidemiology, Economics and Public Health, Royal Veterinary College, London, UK

The article reports the frequency of resistance phenotypes in chicken collected at retail level. The title of the article is appropriate. 

The abstract is clearly written although the consequences of the findings are not clearly referred to. The findings are not really surprising as production methods are bound to impact on the level of resistance among bacteria such as *E. coli.* Also, I think the reference being made to consumers may be misleading as it may imply consumer risk. It would be helpful to provide more details on specific husbandry practices used for kosher chicken as readers may not be familiar with them. 

Regarding the statistical method; I am not clear what the dependent variable for the ANOVA was. It should typically be a numerical not categorical variable, so I assume it was percentage? I understand that analysis was done by brand. So we have a multi-level clustering here (sample-brand-production system). Data should therefore be analysed in this way.

I assume that resistance was established as a binary variable. Note that it is recommended to move towards more quantitative measurement of resistance. The description of the statistical analysis is too superficial to conclude on validity of results. The number of samples was low. 

It has been demonstrated that the extent and type of antimicrobial usage is hugely variable between farms even within one production type (e.g. among conventional producers). It is therefore recommended to use data that allow for linking of resistance status in the product to the true exposure of the animal, i.e. to link retail back to pre-harvest. I know that this is difficult, but else evidence will remain weak.

I have read this submission. I believe that I have an appropriate level of expertise to confirm that it is of an acceptable scientific standard, however I have significant reservations, as outlined above.

# Response

**Published:** 2013-09-17

**Authors:** Bruce Hungate

**Affiliations:** Northern Arizona University, USA

We agree with Dr. Stärk that production methods are likely to impact resistance among generic *E. coli*, as our study shows. Nevertheless, the finding is important, because products available to consumers differ in resistance profiles, according to production practices. The finding that kosher products have higher frequencies of antibiotic resistant *E. coli* is inconsistent with consumers’ perception of kosher as safer and healthier than other options. For this reason, we think the findings are interesting and relevant to consumers.   

Regarding the statistical methods, we appreciate Dr. Stärk’s comments and the opportunity to clarify and provide more information. 

First, the text describing Table 2 in versions 1 and 2 should read, ' *Bold text denotes significant differences among categories according to Chi-square test*'. No ANOVAs were used for binary response variables (in this case, resistant or not resistant). For the test of brand effects, we used ANOVA on non-transformed count data, as reported in the text and in Table 1. We also used the Kruskal-Wallis rank-sum test, which is arguably better suited to count data as it does not require the assumption of a normal distribution (though we did not report those tests). With the Kruskal-Wallis tests, we also found no significant effect of brand within chicken categories. (We will include these results in the **next version**.)  Therefore, we combined brands in our tests comparing drug resistance among categories, as shown in Figure 1B. As identified on the vertical axis in Figure 1B, the dependent variable in the ANOVA was the number of drugs to which strains were resistant (non-transformed). The result is identical using the Kruskal-Wallis rank sum test, which also identified a significant effect of category: chi-squared = 9.3891, df = 3, p-value = 0.02454. 

We also used a general linear model assuming the Poisson distribution which is typical of count data, an approach that allows multiple mean comparisons using the ‘multcomp’ package in R. Tukey’s post hoc tests from the GLM also supported the findings that E. coli from Kosher chicken samples were resistant to more drugs than conventional (P=0.001), organic (P=0.002), and RWA (P<0.001). Thus, our findings are robust and consistent across a number of statistical models.   

We confirm that resistance was defined as a binary variable, as recommended by the Clinical and Laboratory Standards Institute, and as stated in the methods: ‘ *Breakpoint guidelines from the CLSI M100 Tables 2A through 2J for *E. coli *(reference 31) were used to classify strains into "resistant", "intermediate" or "susceptible"; designations of "intermediate" were lumped with "resistant" for purposes of statistics and inference, a conservative approach with respect to consumer safety.*’ Thus, our use of a binary variable for resistance is consistent with the most recent standards for evaluating resistance. 

Nevertheless, we agree with Dr. Stärk that there is merit in considering resistance as a continuous function, and for this reason we provided the raw (continuous) data used to evaluate resistance (see the data depositied on Figshare). Thus, if new standards develop, or if interested readers wish, the raw continuous data are available for analysis.   

We agree with Dr. Stärk that linking the resistance status of the food product to exposure of the animal to antibiotics would provide a stronger basis for causal inference about the source of resistance and identify ways that resistance could be mitigated. However, it would not affect the overall conclusion that production methods yield different frequencies of resistance in *E. coli*, a conclusion that our data clearly support. Obtaining data about on-farm use of antibiotics in the US is nearly impossible at the current time. We hope that analyses like ours help pave the way to greater openness in the industry with respect to the on-farm use of these powerful drugs, and therefore stronger inference about their impacts on food and people.

# Peer review: Referee response for version 1

**Published:** 2013-08-16

**Authors:** Irene Hanning

**Affiliations:** 1 Food Science and Technology, University of Tennessee, Logan, TN, USA

The study is of interest as this type of data is necessary to fully understand the use of antibiotics in animal production. The manuscript is well written and easy to understand. There are a few points that the authors need to address:

**Methods:**

- Please list the number of brands per category that were sampled as in Table 1. Also, please list the number of samples collected per brand. This information would be helpful and may have some impact on the data.

**Results:**

- The total number of *E. coli *isolates collected per type or brand is not stated. It would be helpful to know what percentage of each were positive.

**Discussion:**

- The first sentence of the discussion section is very inflammatory to the industry and is not absolutely true. I would suggest refining this greatly or deleting it.

- Similarly, the second sentence is quite definitive and implies that antibiotic usage always creates antibiotic resistance which may not be true. I suggest modifying this sentence with a qualifying word such as “may select for” or “can select for”.

One variable the authors did not address is the fact that chickens produced by the same brand most likely came from different farms. Because there seems to be a large number of brands sampled, this further adds to the total number of farms that were likely to be sampled. The farm environment does have some impact on the quality of the food. Further, birds from multiple farms may be processed within the same processing plant and this too can impact the microbiological quality of the carcass due to cross-contamination. These are confounding variables that may have impacted the author’s data.

I have read this submission. I believe that I have an appropriate level of expertise to confirm that it is of an acceptable scientific standard, however I have significant reservations, as outlined above.

# Response

**Published:** 2013-09-02

**Authors:** Bruce Hungate

**Affiliations:** Northern Arizona University, USA

We appreciate Dr. Hanning’s comments and suggestions and have revised the manuscript to address each point raised.   

The number of samples collected per brand is now indicated in the text. The number of brands per category is listed as 'N' in Table 1.   

We now state, by type of chicken collected, the percentages of isolates positive for some degree of antibiotic resistance in the fifth sentence of the Results section.  

We have revised a few sentences in the Discussion for clarity and to remove potentially inflammatory or unsupported claims. We have added several citations that provide support for our statements, and at the same time, we removed potentially inflammatory adjectives. We also add a statement about one specific estimate of the extent of antibiotic usage for growth promotion. We realize such estimates are controversial, but we feel that these provide important and useful context for readers in the field. For the second sentence in the original, we have followed Dr. Hanning’s suggestion to modify the claim by changing ' *select*' to ' *can select*'.   

We appreciate the point that farm-to-farm variability could play a role in our results, and have added a sentence acknowledging this explicitly. Also, the third paragraph of the Discussion discusses the potential for cross-contamination within shared production facilities to influence our results. We note that our design was developed to test for significant effects of type of chicken from the perspective of the consumer making decisions about which chicken to purchase. Thus, while farm-to-farm variability and cross-contamination are important potential sources of variation in antibiotic resistance, incorporating this variance is an important part of our design. Future, more exhaustive surveys could attempt to partition the influence of these factors, but doing so was beyond the scope of the current study.

# Peer review: Referee response for version 1

**Published:** 2013-07-23

**Authors:** Marilyn Roberts

**Affiliations:** 1 University of Washington, Seattle, WA, USA

This paper is of interest because it compares chickens raised by conventional, organic, raised without antibiotics, and kosher chickens for the prevalence of antibiotic resistant E. coli.  

The finding that organic and conventionally raised chickens were statically indistinguishable while chickens raised without antibiotics tended to be slightly lower once again raises the question of the tangible and potential health advantages to the consumer of eating organically raised/raised without antibiotic vs. conventionally raised poultry.  Why kosher products had higher prevalence of antibiotic resistant E. coli and E. coli which were multi-resistant is a new finding and certainly needs further study. 

Whether statistical differences in the raised without antibiotic animals would have been found if larger numbers were tested is not clear.  However this study continues to fuel the debate on whether the use of antibiotics as growth promoters in poultry production leads to selection of antibiotic resistant and multi-resistant bacteria; which ultimately may have consequences for treatment of diseases in both man and animals.  This issue has been settled in the EU which has banned the practice, but is of major discussion currently in the US Congress where “The Strategies to Address Antimicrobial Resistance (STAAR)” (which would take important steps to strengthen the US federal response to the public health crisis of antimicrobial resistance) is currently being considered.

I have read this submission. I believe that I have an appropriate level of expertise to confirm that it is of an acceptable scientific standard.

# Response

**Published:** 2013-09-02

**Authors:** Bruce Hungate

**Affiliations:** Northern Arizona University, USA

We very much appreciate Dr. Roberts' comments on our study.
